# Role of PGC-1α signaling in skeletal muscle health and disease

**DOI:** 10.1111/j.1749-6632.2012.06738.x

**Published:** 2012-10-10

**Authors:** Chounghun Kang, Li Li Ji

**Affiliations:** School of Kinesiology, University of MinnesotaMinneapolis, Minnesota

**Keywords:** PGC-1, mitochondria, muscle atrophy, inflammation

## Abstract

This paper reviews the current understanding of the molecular basis of the peroxisome proliferator-activated receptor-γ coactivator-1α (PGC-1α)–mediated pathway and discusses the role of PGC-1α in skeletal muscle atrophy caused by immobilization. PGC-1α is the master transcription regulator that stimulates mitochondrial biogenesis, by upregulating nuclear respiratory factors (NRF-1, 2) and mitochondrial transcription factor A (Tfam), which leads to increased mitochondrial DNA replication and gene transcription. PGC-1α also regulates cellular oxidant–antioxidant homeostasis by stimulating the gene expression of superoxide dismutase-2 (SOD2), catalase, glutathione peroxidase 1 (GPx1), and uncoupling protein (UCP). Recent reports from muscle-specific PGC-1α overexpression underline the importance of PGC-1α in atrophied skeletal muscle, demonstrate enhancement of the PGC-1α mitochondrial biogenic pathway, and reduced oxidative damage. Thus, PGC-1α appears to play a protective role against atrophy-linked skeletal muscle deterioration.

## Introduction

Mitochondria in skeletal muscle tissue experience rapid and characteristic changes as a consequence of manipulations of muscle use and environmental conditions.^1^ Decreased mitochondria content and functional capacity are common muscle pathophysiological traits implicated in the development of mitochondrial myopathy. Skeletal muscle mitochondria are dynamic organelles that play a major role in diverse aspects of cell biology, including ATP production, regulation of intracellular calcium homeostasis, oxidative–antioxidant balance, and apoptosis.^2^ In the past decade, the peroxisome proliferator-activated receptor-γ coactivator-1α (PGC-1α) emerged as a key transcriptional coactivator, which has provided a mechanistic insight for understanding how nuclear regulatory pathways are coupled with the biogenesis of mitochondria, antioxidant defense, and inflammatory response in skeletal muscle. These findings have a profound effect on our understanding of signal transduction pathways related to muscle function and provide new insight into potential new strategies for preventive and therapeutic measures against pathophysiological disorders in skeletal muscle.

## PGC-1α and muscle physiological function

PGC-1α, a transcriptional coactivator, was first identified as a functional activator of the peroxisome proliferator-activated receptor (PPAR)-γ in brown adipose tissue^3^ and is known to influence numerous aspects of metabolism.^4^,^5^ PGC-1α has been identified in other mitochondria-rich tissues, including skeletal, muscle, and heart, as well as in kidney, liver, and brain.^6^ PGC-1α interacts with nuclear receptors and transcription factors to activate transcription of their target genes, and its activity is responsive to multiple stimuli including calcium ion, ROS, insulin, thyroid and estrogen hormone, hypoxia, ATP demand, and cytokines.^7^

### PGC-1α regulation of mitochondrial biogenesis

**Figure 1:**
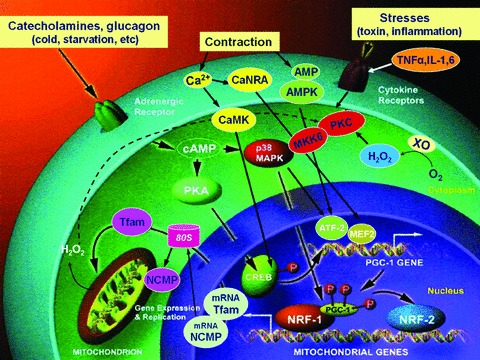
Regulation of PGC-1α gene expression. PGC-1α, peroxisome proliferator-activated receptor gamma coactivator 1-alpha; Tfam, mitochondria transcription factor A; MAPK, mitogen-activated protein kinase; MKK6, MAP kinase kinase: CaMK, calcium/calmodulin-dependent protein kinase; CREB, cyclic AMP response element binding protein; MEF, myocyte enhancer factor; ATF, activating transcription factor; NRF, nuclear respiratory factor; ERR, estrogen-related receptor; XO, xanthine oxidase; PKC, protein kinase C. (Figure modified from Ref. 7.)

Mitochondrial biogenesis is regulated by complex signaling pathways in response to various stimuli. It is a complex process that requires the synthesis, import, and incorporation of proteins and lipids to the existing mitochondrial reticulum, as well as replication of the mitochondrial DNA (mt DNA).^8^ PGC-1α has been thought to be a master regulator of mitochondrial biogenesis by coactivating several transcription factors that, in turn, bind to the promoters of distinct sets of nuclear-encoded mitochondrial genes.^2^,^9^ It is known that PGC-1α interacts with several nuclear transcription factors, including PPAR family members, nuclear respiratory factor (NRF)-1 and NRF-2, and estrogen-related receptor-α (ERR-α), as well as myocyte enhancer factor-2 (MEF2), forkhead box protein O (FOXO) 1, and sterol regulatory element-binding proteins (SREBP) 1.^10^,^11^ For example, PGC-1α coactivation of NRF-1, 2 promotes the expression of numerous nuclear-encoded mitochondrial proteins (NEMP), as well as mitochondrial transcription factor A (Tfam), which directly stimulates mitochondrial DNA (mtDNA) replication and transcription (Fig. 1).^7^,^12^,^13^ Overexpression of the PGC-1α in cultured myoblasts and other cells induces respiratory subunit mRNAs and increases cytochrome c oxidase subunit 4 (COXIV) and cytochrome *c* (Cyt C) protein levels as well as the steady-state level of mitochondria DNA (mtDNA),^14^ as an adaptation to facilitate increased oxygen utilization. In skeletal muscle, PGC-1α has also been shown to regulate skeletal muscle fiber type switch, glucose transport, and lipid utilization,^15^–^17^ as well as mitochondrial biogenesis and fusion.^3^,^18^

PGC-1α–driven mitochondrial biogenesis is tissue specific and developmental stage dependent. Cardiac-specific overexpression of PGC-1α during the neonatal stages leads to a dramatic increase in cardiac mitochondrial number and size coincident with upregulation of gene markers associated with mitochondrial biogenesis, whereas in adult mice there is only a modest increase in mitochondrial number, associated with derangements of mitochondrial ultrastructure and development of cardiomyopathy.^19^ Previously, similar adverse effects following PGC-1α overexpression were also reported for the heart. Cardiac-specific overexpression of PGC-1α in transgenic mice resulted in uncontrolled mitochondrial proliferation in cardiac myocytes leading to loss of sarcomeric structure and a dilated cardiomyopathy.^20^ Furthermore, an increased mitochondrial respiratory capacity is intimately linked to an increased production of ROS that may further repress survival pathways, hence promoting apoptosis signaling.^21^ The increased number of mitochondria in PGC-1α–overexpressing neurons influences the axonal transport of these organelles thereby perturbing their normal turnover.^22^

### Role of PGC-1α in antioxidant enzymes

Recent studies have shown that PGC-1α also has a regulatory mechanism for the expression of endogenous antioxidant proteins. Reduced mRNA levels of SOD1 (CuZn-SOD), SOD2 (Mn-SOD), and/or GPx1,^23^ as well as SOD2 protein content,^24^,^25^ were observed in skeletal muscle from PGC-1α knockout (KO) mice compared to wild type (WT), while PGC-1α overexpression mice showed an upregulation of SOD2 protein content in skeletal muscle.^26^ PGC-1α KO fibroblasts exhibit a decrease in SOD2, catalase, and GPx1 mRNA content relative to WT fibroblasts and PGC-1α KO mice were more vulnerable to oxidative stress.^27^ In addition, PGC-1α has been shown to regulate RNA expression of UCP 2 and 3 in cell culture,^28^ suggesting that PGC-1α may also increase the uncoupling capacity and concomitantly reduce mitochondrial ROS production. Furthermore, it has been also shown that PGC-1α promotes mSIRT3 gene expression, which is mediated by an ER-α binding element mapped to the SIRT3 promoter region.^29^ SIRT3 binds to, deacetylates, and activates mitochondrial enzymes, including SOD2, through a posttranslational mechanism.^30^,^31^ Taken together, PGC-1α seems to have a role in reducing ROS damage by upregulating antioxidant gene expression and activity.

### Role of PGC-1α in inflammation

Recent observations further indicate that PGC-1α may also play a role in antiinflammatory effects. Studies in PGC-1α KO animals indicated that PGC-1α modulates local or systemic inflammation and might regulate the expression of inflammatory cytokines and inflammatory markers such as TNF-α and IL-6.^32^,^33^ PGC-1α KO mice showed higher basal mRNA expression of TNF-α, IL-6 in skeletal muscle, as well as higher serum IL-6 levels than WT.^2^ In addition, PGC-1α–overexpressed mice had lower expression of TNF-α and IL-6 mRNA in skeletal muscle, and overexpression of PGC-1α reduced an age-associated increase in TNFα and IL-6 protein content in skeletal muscle and reduced serum TNF-α and IL-6 levels in old mice compared with age-matched WT mice.^26^ These data suggest that PGC-1α has a protective role in inflammatory response by reducing proinflammatory cytokine production. Moreover, a single exercise bout elicited a significant increase in skeletal muscle TNF-α mRNA and serum TNF-α content in PGC-1α KO mice, but not in WT mice,^2^ indicating that skeletal muscle PGC-1α normally protects against exercise-induced increases in TNF-α. These findings suggest that the transcriptional regulation by which muscle cells produce these cytokines are enhanced by the recruitment of particular immune cells (polymorphoneutrophil, macrophage), which inflicts an inflammatory response to the muscle stress and damage, whereas PGC-1α usually functions to suppress the production of such inflammatory factors.^2^

Although the underlying mechanism that links the PGC-1α–mediated anti-inflammatory effect is still not known, PGC-1α–mediated regulation of the antioxidant defense can be a significant determinant as increased oxidative stress due to an impaired balance between ROS production and removal can induce an inflammatory response through activation of the redox sensitive transcription factor, NF-κB. NF-κB is a ubiquitous signaling pathway that is involved in the pathology of inflammatory diseases.^34^ The NF-κB pathway is activated by phosphorylation, ubiquitination, and proteolysis of the protein inhibitory kappa B (IκB), which binds and retains the NF-κBp65/p50 heterodimer in the cytosol. Following an activation signal, such as TNF-α, inhibitory kappa B kinase (IKK) phosphorylates IκB, resulting in the protein's ubiquitination and proteasome degradation. After the destruction of IκB, p50, and p65 are released and enter the nucleus to affect gene transcription of various proteins involved in immune function, inflammatory response, and antioxidant defense.^35^

Previous research has shown that ROS induce inflammatory cytokine production in skeletal muscle,^36^ and the expression of mitochondrial ROS-detoxifying enzymes was increased by PGC-1α.^28^,^37^ Downregulation of the antioxidant genes in muscle-specific PGC-1α KO mice increased cytokine expression.^38^,^39^ These studies suggest that in parallel with increasing mitochondrial respiration PGC-1α is a suppressor of ROS production through the PGC-1α–mediated upregulation of antioxidant enzymes, as well as UCPs that also attenuate ROS production by changing electron transport chain redox status.^28^,^37^

## Effect of exercise on PGC-1α expression and mitochondrial biogenesis

### Role of PGC-1α in mitochondrial adaptation to exercise

It is well known that endurance training can increase mitochondrial content and respiratory capacity in skeletal muscle,^40^ resulting in a slower rate of utilization of muscle glycogen and blood glucose, a greater reliance on fat oxidation, and less lactate accumulation during submaximal exercise.^41^ PGC-1α is known to be a major regulator of exercise-induced phenotypic adaptation and fiber transformation from type 2 to type 1.^4^ In skeletal muscle, PGC-1α expression is linked to muscle contraction through Ca^2+^/calmodulin-dependent protein kinase IV (CamKIV). It is known that CamKIV and calcineurinA are activated through calcium ion dynamics within the muscle in response to exercise.^42^ The increased calcium signaling during muscle contraction activates several important transcription factors, such as cAMP-response element binding protein (CREB), which is a target of CamKIV, and myocyte enhancer factors (MEF) 2.^43^ Another factor that regulates PGC-1α expression upon exercise involves p38 MAPK, which activates MEF2 and activating transcription factor 2 (ATF2). p38 MAPK, in conjunction with ATF2, results in increased expression of PGC-1α.^44^ ATF2 and subsequent interactions of ATF2-CREB appeared to be an early event in PGC-1α–mediated signaling processes.^45^ p38 MAPK also stimulates PGC-1α by phosphorylation in response to cytokine stimulation in muscle cells.^46^ Finally, as a metabolic energy deprivation sensor, AMPK is activated by exercise due to increased AMP/ATP ratio and Ca^2+^ flux during muscle contraction, enhancing PGC-1α transcription as well as activity. It was demonstrated that activation of p38 MAPK-mediated phosphorylation of subsequent CREB binding to the PGC-1α promoter plays a key role in activating PGC-1α expression in response to increased muscle activity.^47^

**Figure 2:**
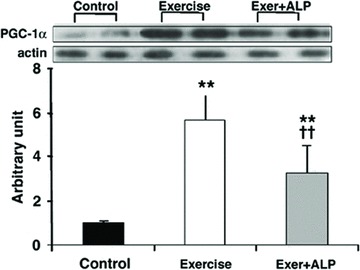
Western blot analysis of PGC-1α and actin protein contents in rat deep vastuslateralis (DVL) muscle. Data were expressed as the ratio of densitometric intensity of PGC-1α and actin protein bend. Exer, treadmill running for 88 minutes; ALP, allopurinol injection 1 and 24 h prior to exercise. **P < 0.01, exer or exer + ALP versus control; ++*P* < 0.01, exer + ALP versus exer.

Endurance exercise is known to be a powerful stimulus to muscle plasticity, such as a fiber-type switching toward more oxidative fibers.^4^,^43^ PGC-1α KO mice exhibit a shift from oxidative to glycolytic muscle fibers. Moreover, skeletal muscle-specific PGC-1α KO animals have reduced endurance capacity and exhibit fiber damage and elevated markers of inflammation following treadmill running.^38^ We further examined the redox-sensitive nature of PGC-1α signaling during acute sprinting exercise and reported that reducing ROS generation with allopurinol to inhibit xanthine oxidase (XO), the main ROS source of this type of exercise, attenuated PGC-1α expression and PGC-1α–controlled signaling pathway and mitochondrial biogenesis (Fig. 2).^48^

### PGC-1α and age-associated mitochondrial dysfunction: effect of exercise

Loss of skeletal muscle mass and strength during aging^26^ and lack of exercise are thought to be significant risk factors in developing sarcopenia.^49^ Transcriptional profiles of skeletal muscle show a decrease in the expression of mitochondrial genes with age^50^ and result in an age-related accumulation of old, damaged, or impaired mitochondria by the reduction of mitochondria turnover.^51^ Aging is known to alter several important mechanisms that may affect PGC-1α expression. For example, ROS generation is increased in aged skeletal muscle, which activates NFκB, a negative regulator of PGC-1α.^52^

**Figure 3:**
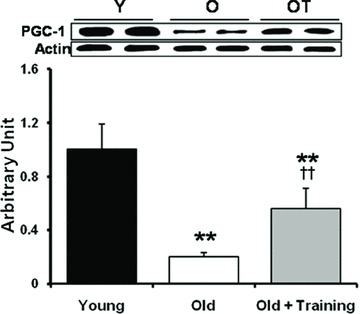
Aging affects of PGC-1α on rat soleus muscle. Twelve weeks of endurance training restores PGC-1α protein contents. Western blot detection of PGC-1α normalized with actin protein content. Each bar represents mean ± SEM (*N*= 3). **P < 0.01; young (Y) versus old (O) or old training (OT), ††*P* < 0.01; old (O) versus old training (OT). Three groups of Fischer 344/BNF1 rats (young, rested (Y, four months old, *N*= 3); old, rested (O, 24 months old, *N*= 3); and old, subjected to a 12-week treadmill running at 17.5 m/min, 10% grade for 45 min/day five days/week (OT, 24 months old, *N*= 3)).

We recently investigated whether exercise training can ameliorate age-associated decline of PGC-1α gene expression and mitochondrial biogenesis.^53^ The PGC-1α mRNA level was decreased 35% in the soleus muscle of old rats but was restored by training. PGC-1α protein content was 80% lower in old rats than young rats, whereas 12 weeks of exercise training resulted in a 2.7-fold higher PGC-1α content in the old rats (Fig. 3). Mitochondria are important regulators of Ca^2+^ homeostasis; its function can be regulated by the Ca^2+^-sensitive pathway, for example, via CREB. Phospho-CREB (p-CREB) content was 50% lower in old rats compared to young rats, but endurance training increased p-CREB content significantly. Furthermore, CREB binding activity assessed by electromobility shift assay was lowered in old rats, whereas its level was significantly increased after endurance training in old rats.

As the mitochondrial biogenesis markers, both mRNA and protein contents of Tfam and Cyt. c in the soleus muscle were lower in the old control rats whereas endurance training significantly elevated Tfam and Cyt. c protein levels (data not shown).

The ratio of AMP:ATP is a sensor of muscle energy status and an increased AMP:ATP ratio can lead to AMPK activation and upregulation of PGC-1α. AMPK protein contents were decreased in old rats and there was no effect of endurance training on AMPK content. However, endurance training increased p-AMPK content in old rats (data not shown). Thus, these data indicate that endurance training can attenuate aging-associated decline in PGC-1α–activated mitochondrial biogenesis in skeletal muscle.

## Effect of PGC-1α overexpression on muscle disuse atrophy

### Pathways of skeletal muscle atrophy

Muscle atrophy caused by prolonged immobilization (IM) appears to be a highly ordered and regulated process, which is characterized by decreased cross-sectional muscle fiber area, reduced force production, increased fatigability, and insulin resistance.^54^ These alterations also include decreased protein synthesis, increased oxidative stress, increased protein degradation, and suppression of biogenesis associated with mitochondrial dynamics.^55^ Different signaling pathways may be involved in causing muscle atrophy depending on the upstream perturbations, such as decreased IGF1-AKT-FoxO signaling, inflammatory cytokines and NF-κB signaling, and reduced nutrition and energy input. During muscle IM, atrophy is associated with a common transcriptional profile and activation of the ubiquitin-proteasome pathway. Imbalance of ROS production and antioxidant defense resulting in oxidative stress plays an important role in protein breakdown in skeletal muscle during periods of inactivity.^56^ New evidence suggests that mitochondria may be an important source of ROS production in inactive muscle.^57^ A downregulation of PGC-1α was observed in muscle atrophy of different models and thought to be a major molecular mechanism for enhanced FoxO phosphorylation, NF-κB activation, and protein loss.^58^ Indeed, PGC-1α KO mice displayed higher basal expression of TNF-α and IL-6 than WT.^38^,^59^ Conversely, transgenic mice with PGC-1α overexpression have been shown to have a decreased inflammatory cytokine production and protein degradation due to denervation.^26^,^58^

### Protective role of PGC-1α in disuse atrophy

Previous studies suggest that PGC-1α has a protective role against protein catabolism and muscle wasting in a variety of contexts. For instance, denervation-induced muscle atrophy and the effects of Duchenne's muscular dystrophy are greatly ameliorated when the amount of PGC-1α is maintained at normal levels or increased.^58^,^60^ However, adverse effect of PGC-1α on disuse atrophy seems to occur on fast type muscle fibers. It has been reported that overexpression of PGC-1α in mouse skeletal muscle increased uncoupled oxidative phosphorylation and caused muscle atrophy following ATP depletion in fast fiber type (type IIb).^61^ Conversely, activation of the PGC-1α pathway in postnatal dystrophic (mdx) mice, induced expression of proteins associated with slow fiber type, which are more resistant to contraction-induced damage.^62^

**Figure 4:**
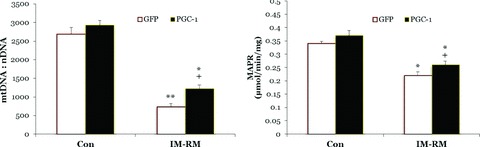
Effect of PGC-1α overexpression on mitochondria content and function in mice atrophied muscles. Mitochondria DNA content (mtDNA/nDNA) and ATP production rate (MAPR) were determined in the FVB/N mice TA (tibialis anterior) muscles. Data are mean + SEM. *P < 0.05 IM-RM versus control; **P < 0.01 IM-RM versus control; +*P* < 0.05 PGC-1α versus GFP. IM-RM, 14 days immobilization plus 5-day remobilization.

Inactivity-induced deficit of PGC-1α in skeletal muscle results in a chronic systemic inflammatory state, which has serious pathological consequences. Interestingly, restoration of muscle movement (RM) resulted in an initial increase in ROS generation, proinflammatory cytokine expression, and oxidative stress, which all prevent muscle recovery and prolongs functional impairment of performance.^63^ Using a mouse hind limb IM followed by RM model, we have recently found that overexpression of PGC-1α via local electroporation and transfection increased mitochondrial biogenesis and ATP production, lowered inflammatory cytokine TNF-α and IL-6 expression, and reduced NF-κB activation in response to IM-RM. These improvements were accompanied by less ROS generation and oxidative stress, and elevated SOD2 activity. As Figure 4 shows, PGC-1α–overexpressed mice had significantly higher mitochondrial (mt)DNA content and ATP production compared to their empty vesicle injected controls after a 19-day IM-RM regimen.

## Conclusion

Although it has only been over a decade since PGC-1α was first discovered, this versatile and master transcriptional regulator has receive tremendous attention among scientists from many different fields. Undoubtedly, PGC-1α plays a critical role in maintaining muscle metabolic function and controls numerous genes that affect a broad range of muscle morphology and physiological function. Inactivity and aging are two important negative factors that downregulate PGC-1α gene expression and subsequent downregulation of mitochondrial biogenesis. Physical exercise, among other physiological stimulators, can activate PGC-1α expression through several signal transduction pathways. This adaptation may explain a majority of previously reported endurance training effects on muscle. Thus, overexpression of PGC-1α level in muscle via nonexercise means may be an attractive alternative to restore and promote muscle metabolic function under the situation when normal physical activity is impossible.
